# Identification of the Highly Polymorphic Prion Protein Gene (*PRNP*) in Frogs *(Rana dybowskii*)

**DOI:** 10.3390/ani15020220

**Published:** 2025-01-15

**Authors:** Chang-Su Han, Sae-Young Won, Sang-Hun Park, Yong-Chan Kim

**Affiliations:** Department of Biological Sciences, Andong National University, Andong 36729, Republic of Korea; 20180701@student.anu.ac.kr (C.-S.H.); gkfh32@staff.anu.ac.kr (S.-Y.W.); 20245018@student.anu.ac.kr (S.-H.P.)

**Keywords:** amyloid, bovine spongiform encephalopathy, Creutzfeldt–Jakob disease, chronic wasting disease, ecosystem, frog, prion, *PRNP*, scrapie, single nucleotide polymorphism, susceptibility

## Abstract

Prion diseases are fatal neurodegenerative diseases that can be transmitted by infectious protein particles, PrP^Sc^s, encoded by the endogenous prion protein gene (*PRNP*). The origin of prion seeds is unclear, especially in non-human hosts, and this identification is pivotal to preventing the spread of prion diseases from host animals. Recently, an abnormally high amyloid propensity in prion proteins (PrPs) was found in a frog, of which the genetic variations in the *PRNP* gene have not been investigated. In this study, genetic polymorphisms in the *PRNP* gene were investigated in 194 Dybowski’s frogs using polymerase chain reaction (PCR) and amplicon sequencing. We carried out in silico analyses to predict functional alterations according to non-synonymous single nucleotide polymorphisms (SNPs) using PolyPhen-2, PANTHER, SIFT, and MutPred2. We used ClustalW2 and MEGA X to compare frog *PRNP* and PrP sequences with those of prion-related animals. To evaluate the impact of the SNPs on protein aggregation propensity and 3D structure, we utilized AMYCO and ColabFold. We identified 34 novel genetic polymorphisms including 6 non-synonymous SNPs in the frog *PRNP* gene. The hydrogen bond length varied at codons 143 and 207 according to non-synonymous SNPs, even if the electrostatic potential was not changed. In silico analysis predicted S143N to increase the aggregation propensity, and W6L, C8Y, R211W, and L241F had damaging effects on frog PrPs. The *PRNP* and PrP sequences of frogs showed low homology with those of prion-related mammals. To the best of our knowledge, this study was the first to discover genetic polymorphisms in the *PRNP* gene in amphibians.

## Simple Summary

Prion diseases are fatal neurodegenerative diseases that can be transmitted by infectious protein particles, PrP^Sc^s, encoded by the endogenous prion protein gene (*PRNP*). In a recent study, an abnormally high amyloid propensity in prion proteins (PrPs) was observed in a frog; however, genetic polymorphisms in the *PRNP* gene have not been investigated thus far. In the present study, we found 34 novel genetic polymorphisms including 6 non-synonymous SNPs in the frog *PRNP* gene. The hydrogen bond length varied at codons 143 and 207 according to the non-synonymous SNPs. In addition, S143N was predicted to increase aggregation propensity, while W6L, C8Y, R211W, and L241F were predicted to have damaging effects on the frog PrP. This was the first genetic study of genetic polymorphisms in the *PRNP* gene in amphibians.

## 1. Introduction

Prion diseases are neurodegenerative disorders that occur in the brain due to the accumulation of misfolded prion protein (PrP^Sc^), which is an infectious protein converted from normal prion protein (PrP^C^) [1,2]. PrP^Sc^ has a proteinase-K-resistant structure that is richer in beta sheets and poorer in alpha helix compared to PrP^C^ [3]. Prion diseases have been reported among several mammalians. In humans, there are several types of prion diseases including the Creutzfeldt–Jakob disease (CJD), Kuru, Gerstmann–Sträussler–Scheinker syndrome (GSS), and fatal familial insomnia (FFI) [4]. In addition, there are several types of non-human prion diseases including scrapie in sheep and goats, bovine spongiform encephalopathy (BSE) in cattle, and chronic wasting disease (CWD) in elk and deer [5].

PrP^C^, a template of PrP^Sc^, is translated from the prion protein gene (*PRNP*), the genetic polymorphisms of which can change the protein structure of the prion protein (PrP) and serve a key role in susceptibility/resistance to prion diseases in susceptible animals [5,6,7]. In sheep, the susceptibility to scrapie is modulated by genetic variations in the ovine *PRNP* gene including A136V, R154H, and Q171R. Haplotypes in the ovine *PRNP* gene at codons 136, 154, and 171 are significantly related to the risk of scrapie, and the variations V136, R154, and Q171 (VRQ haplotypes) are the most scrapie-sensitive haplotype [8,9]. In goats, I142M, H143R, N146S, R154H, R211Q, and Q222K polymorphisms significantly increase the resistance to scrapie [10,11]. In cattle, 12 bp and 23 bp insertion/deletion polymorphisms located in the regulatory region of the bovine *PRNP* gene are linked to susceptibility to BSE, and the highest-risk haplotypes to BSE are 12 bp and 23 bp deletion alleles [12,13]. In elk, individuals with the M132L genotype are susceptible to prion disease [14]. In addition, Q95H, G96S, and A116G are associated with susceptibility to CWD in white-tailed deer [15,16,17].

During the BSE pandemic, it became evident that prion-contaminated meat and bone meal was fed to cattle, and the resulting infected beef was subsequently consumed by humans [18,19,20]. There is a hypothesis that the prion originated from scrapie, although the origin of scrapie remains unknown [21]. Given that pathogenic single nucleotide polymorphisms (SNPs) have been reported only in humans and that prion seed SNPs have not been reported in non-human prion host animals, prion seeds derived from animals at lower evolutionary stages may be the cause of scrapie. Because PrP^Sc^ is highly resistant to degradation in the environment, there is a possibility that prion seeds were transmitted to sheep and goats by contaminated soil, water, or plants [22,23,24]. Amphibians occupy an important niche in ecosystems, travelling between water and land, feeding on insects and small invertebrates, and acting as prey to various predators [25,26]. Recently, Won et al. found that a certain type of frog PrP exhibits an abnormally high aggregation propensity [27]. Moreover, their findings suggest that specific SNPs in frog PrP significantly increase the likelihood of prion formation. These results underscore the importance of investigating genetic variations in the frog *PRNP* gene to assess the potential for prion seed formation. Dybowski’s frog is a native species of Korea, usually distributed in Northeast Asia, throughout the Republic of Korea [28], and has a unique feature among frog species in that it generally inhabits a woodland environment except during breeding [29,30]. This frog is widely consumed as a health food in the Republic of Korea. Given the possibility of direct transmission of prion seeds to humans, studies on *PRNP* polymorphisms in Dybowski’s frog could be necessary.

In the present study, we investigated *PRNP* polymorphisms using polymerase chain reaction (PCR) and amplicon sequencing in Dybowski’s frogs (*Rana dybowskii*). In addition, we predicted the secondary and tertiary structures of the frog PrP according to non-synonymous SNPs using ColabFold Ver. 1.5.5 and Swiss-Pdb viewer Ver. 4.1.0 software [31,32]. Furthermore, we evaluated the impact of non-synonymous SNPs on frog PrP in silico using AMYCO, PolyPhen-2, PANTHER, MutPred2, and SIFT [33,34,35,36,37]. Lastly, we performed multiple sequence alignments using ClustalW2 [38] and constructed phylogenetic trees with the MEGA X program [39] for frog *PRNP* and PrP sequences, comparing them with those from prion-related animals to elucidate their evolutionary relationships.

## 2. Materials and Methods

### 2.1. Animal Samples

A total of 194 Dybowski’s frogs (*Rana dybowskii*) were obtained from frog farms in the Republic of Korea. Detailed information on the farm locations is provided in Supplementary Table S1.

### 2.2. Genomic DNA Extraction

Genomic DNA was extracted from the tissue (~20 mg) of each muscle sample using a Da-bead^TM^ Genomic DNA prep kit (BIOFACT, Daejeon, Republic of Korea) following the described protocol.

### 2.3. PCR

We customized forward (*PRNP* gene_F: 5′-TTGCAAAATGAGACCCTGTG-3′, 20mer) and reverse (*PRNP* gene_R: 5′-GCAGTGGAGCAGGATATCTGTAT-3′, 23mer) sequence-specific primers from the GenBank (Gene ID: 120932994) database to amplify open reading frame (ORF) regions in exon 2 of the *PRNP* gene (759 bp). Then, PCR was performed with 25 μL of master mix, using a BioFACT™ Taq DNA Polymerase Kit (BIOFACT, Daejeon, Republic of Korea) according to the manufacturer’s protocol. The PCR master mix consisted of 2.5 μL of 10× Taq Buffer, 1 μL of F_primer (10 pmol), 1 μL of R_primer (10 pmol), 0.5 μL of dNTP, 0.125 μL of Taq DNA Polymerase (5 U/μL), 17.875 μL of DW, and 2 μL of genomic DNA (20–30 ng/μL). Genome amplification was carried out using a T-100 Thermal Cycler (BIO-RAD, Hercules, CA, USA). The conditions of PCR were pre-denaturation at 95 °C for 2 min, 34 cycles of denaturation at 95 °C for 30 sec, 60 °C annealing for 30 s, and 72 °C extension for 1 min, followed by a final extension at 72 °C for 5 min. The PCR product was loaded in 6× loading buffer (Takara™, Kusatsu, Japan) and separated in a 1% agarose gel stained with GreenStar™ Nucleic Acid Staining Solution I (BIONEER, Daejeon, Republic of Korea) using electrophoresis (100 V, 60 min). The target PCR band was clearly observed using a UV Transilluminator (DAIHAN Scientific, Wonju, Republic of Korea) and cut from the gel for elution with an AccuPrep^®^ PCR/Gel Purification Kit (BIONEER, Daejeon, Republic of Korea) for amplicon sequencing using an ABI PRISM 3730XL Analyzer (ABI, Foster City, CA, USA).

### 2.4. Genotyping

The electropherograms of amplicon sequencing results were analyzed using Finch TV software Ver. 1.4.0 (Geospiza Inc, Seattle, WA, USA), and genotyping was performed using ApE software https://jorgensen.biology.utah.edu/wayned/ape/ (accessed on 11 July 2023) to identify genetic polymorphisms. Genotyping was confirmed using both forward and reverse primers, and only files with a Q > 30 value were utilized for analysis. Quality control (QC) procedures were conducted based on the Hardy–Weinberg Equilibrium.

### 2.5. In Silico Analyses

The aggregation propensity of frog PrP according to non-synonymous SNPs was evaluated by AMYCO software. The AMYCO score exists in the range of 0.0 to 1.0, with values closer to 0.0 representing a lower aggregation tendency and those closer to 1.0 indicating a higher aggregation tendency. Functional alterations according to non-synonymous SNPs of the *PRNP* gene were evaluated by using PolyPhen-2 Ver. 2.2.3, PANTHER Ver. 19.0, MutPred2 Ver. 2.0, and SIFT Ver. 6.2.1 software. PolyPhen-2 predicts the possible impact of amino acid substitutions on the structure and function of proteins using straightforward physical and evolutionary comparative considerations. PolyPhen-2 presents 3 results: “Benign”, “Possibly Damaging”, or “Probably Damaging” based on pairs of false positive rate (FPR) thresholds between 0 and 1. PANTHER represents the effect on the protein function as “Probably Benign”, “Possibly Damaging”, “Probably Damaging” comparing the entered protein sequence with that in the PANTHER protein library. The position-specific evolutionary preservation (PSEP) score comes out with the PANTHER result. It calculates the time (millions of years) that the amino acid has been preserved. The longer the preservation time, the higher the likelihood of a negative alteration in protein function. The PSEP less than 200 million years (my) is “Probably Benign”, between 200 my and 450 my is “Possibly Damaging”, and greater than 450 my is “Probably Damaging”. The output of MutPred2 consists of a general score and property score. The general score ranges between 0 and 1, with a score higher than 0.5 indicating “Pathogenic,” and a lower score indicating “Neutral” [40]. A general score higher than 0.5 for MutPred2 indicates that it is pathogenic and that there are structural or functional changes in the protein. For the property score, a higher value is more likely to represent a variant. The SIFT score ranges from 0 to 1, with scores ≤0.05 reported as “Damaging”, while scores >0.05 predict a substitution to be “Tolerated” [33].

### 2.6. Statistical Analysis

We analyzed linkage disequilibrium (LD) and haplotype distributions of the 34 polymorphisms with HaploView software version 4.2 (Broad Institute, Cambridge, MA, USA).

### 2.7. Three-Dimensional Structure Analysis

As hydrogen bond length and electrostatic potential in a protein structure can change depending on amino acid variants, we analyzed 3D structures using ColabFold Ver. 1.5.5: AlphaFold2 using MMseqs software based on protein sequences. Subsequently, we predicted whether hydrogen bond length and electrostatic potential would change depending on non-synonymous SNPs using the Swiss-Pdb viewer program. Hydrogen bonds were predicted based on atom distance, atom type, and angle. If the Pdb file included H atom coordinates, bonds were identified within a 1.2–2.76 Å range from the donor atom. Bonds within these ranges are shown as green dotted lines, while weaker bonds exceeding the distance by 0.05 Å are displayed as gray dotted lines.

### 2.8. Multiple Sequence Alignments and Phylogenetic Analysis

The DNA and amino acid sequences of PrPs were obtained from GenBank. Detailed information on the sequences is as follows: *Homo sapiens* (human, Gene ID: 5621, Protein ID:NP_001073590.1), *Ovis aries* (sheep, Gene ID: 493887, Protein ID: XP_060252821.1), *Capra hircus* (goat, Gene ID: 102169975, Protein ID: XP_005688214.2), *Bos taurus* (cattle, Gene ID: 281427, Protein ID: NP_851358.2), *Odocoileus hemionus* (mule deer, Gene ID: MT710106.1, Protein ID: QMT15698.1), *Canis lupus familiaris* (dog, Gene ID: 485783, Protein ID: NP_001013441.1), *Equus caballus* (horse, Gene ID: 100065904, Protein ID: NP_001137270.2), *Rana dybowskii* (Dybowski’s frog, Gene ID: In this study, Protein ID: in this study). Multiple sequence alignments were performed using ClustalW2 https://www.ebi.ac.uk/Tools/msa/clustalo/ (accessed on 29 December 2024). The phylogenetic analysis of amino acid sequences of PrPs was performed using the MEGA X program [39] with the neighbor-joining method (3000 bootstrap replicates). Evolutionary distances were calculated using the Poisson correction method and visualized through a phylogenetic tree.

## 3. Results

### 3.1. Identification of the Novel Polymorphisms in the Frog PRNP Gene in 194 Dybowski’s Frogs

**Figure 1:**
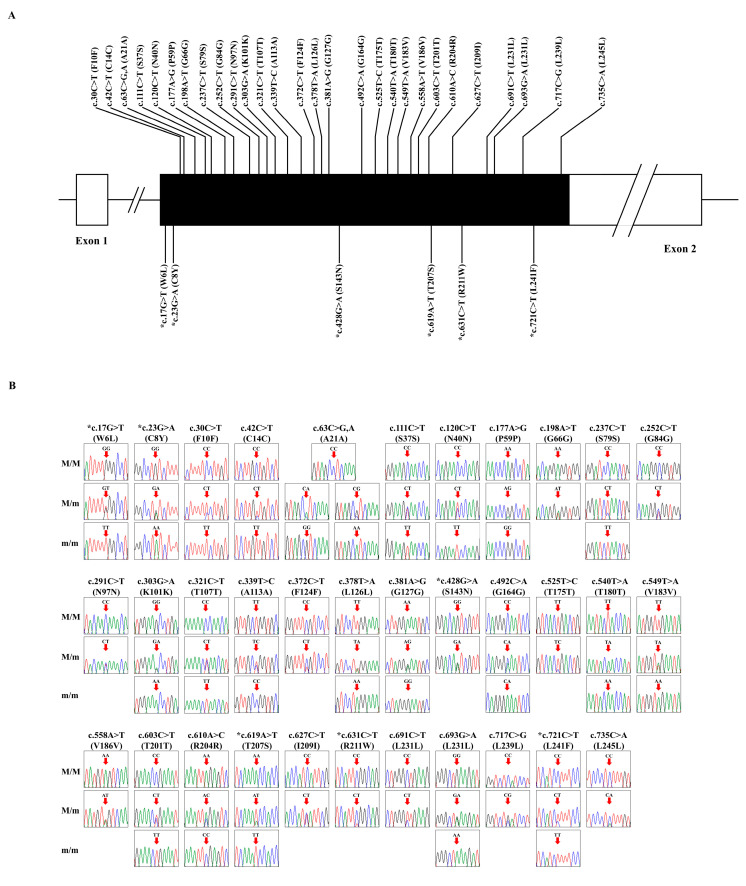
Identification of 34 novel single nucleotide polymorphisms (SNPs) of the frog prion protein gene (*PRNP*) found in this study. (**A**) The diagram describes the frog *PRNP* gene. In exon 2, the open reading frame (ORF) is represented by the black box. The white boxes depict non-coding exons. On the black box, the SNPs shown above are synonymous SNPs, while the SNPs shown below (marked with an asterisk) are non-synonymous. (**B**) Electropherograms describe the 34 novel SNPs discovered in the frog *PRNP* gene. Non-synonymous SNPs are indicated with an asterisk. The peaks in the box indicate each base of DNA sequence as follows: green: adenine; black: guanine; blue: cytosine; red: thymine. The red arrows indicate the locations of SNP sites. M/M: major allele homozygote; M/m: heterozygote; m/m: minor allele homozygote.

The frog *PRNP* gene consists of 2 exons, and exon 2 includes the ORF. We designed frog *PRNP* gene sequence-specific primers based on the *PRNP* sequence of a common frog (*Rana temporaria*) registered in GenBank (Gene ID: 120932994). We performed amplicon sequencing of the *PRNP* gene and found 34 novel SNPs including c.17G>T, c.23G>A, c.30C>T, c.42C>T, c.63C>G,A, c.111C>T, c.120C>T, c.177A>G, c.198A>T, c.237C>T, c.252C>T, c.291C>T, c.303G>A, c.321C>T, c.339T>C, c.372C>T, c.378T>A, c.381A>G, c.428G>A, c.492C>A, c.525T>C, c.540T>A, c.549T>A, c.558A>T, c.603C>T, c.610A>C, c.619A>T, c.627C>T, c.631C>T, c.691C>T, c.693G>A, c.717C>G, c.721C>T, and c.735C>A (Figure 1). Among these, c.17G>T (W6L), c.23G>A (C8Y), c.428G>A (S143N), c.619A>T (T207S), c.631C>T (R211W), and c.721C>T (L241F) were non-synonymous SNPs. Notably, the c.63C>G,A polymorphism was trimorphic. The genotype and allele frequencies for all polymorphisms are described in Table 1.

### 3.2. Haplotype Analysis of the Frog PRNP Polymorphisms

To analyze the haplotype frequencies of 34 novel polymorphisms, we carried out a haplotype analysis using HaploView Ver. 4.2 software. We identified 19 major haplotypes, 3 of which were dominant haplotypes with greater than 8% frequency: GGCCGCCAACCCGCTCTAGCTTTACAACCCGCTC (12.6%), TGCCCCTAACCCGCTCTAGCTTTACAACCCGCCC (11.1%), and GGCCCCCAACCCGCTCTAGCTTTACAACCCGCCC (8.7%). The detailed results are described in Table 2.

### 3.3. LD Analysis Among the 34 Polymorphisms in the Frog PRNP Gene

**Figure 2:**
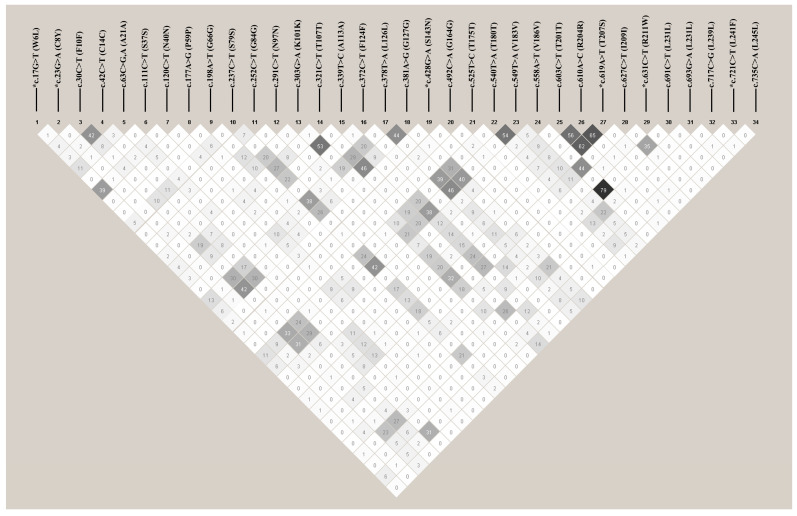
The linkage disequilibrium (LD) block structure consisting of 34 SNPs located in the frog *PRNP* gene. The coefficient of the LD (r^2^ value) between the SNPs was calculated by HaploView Ver. 4.2 software. The LD color scale ranges from white to black, with an increasing color intensity corresponding to higher r² values.

We performed an LD analysis of 34 novel frog *PRNP* polymorphisms using HaploView Ver. 4.2 software. Among the 34 polymorphisms, the 20 with strong LD values were estimated. The strongest LD was identified between c.558A>T and c.691C>T, followed by c.610A>C and c.619A>T, c.603C>T and c.619A>T, and c.603C>T and c.610A>C. The detailed values are described in Figure 2.

### 3.4. Artificial-Intelligence-Based Prediction of the 3D Structure of the Frog PrP According to Non-Synonymous SNPs

**Figure 3:**
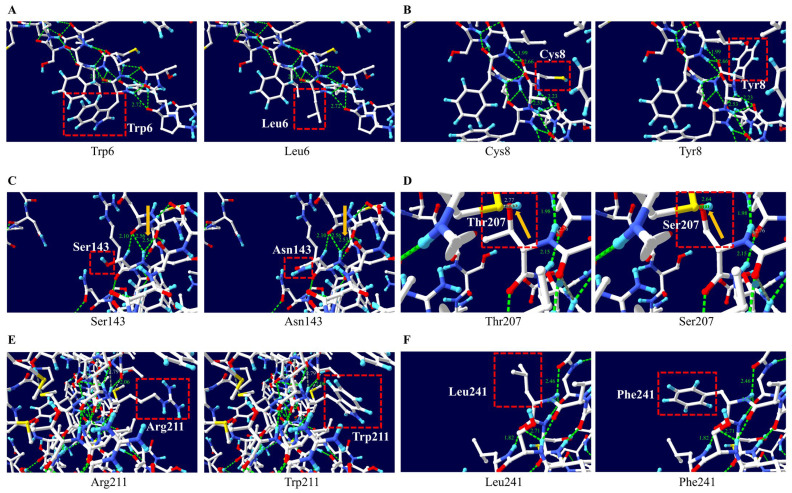
The analysis of the hydrogen bond alterations in the frog prion protein (PrP) according to 6 non-synonymous SNPs was evaluated using Swiss-Pdb Viewer Ver. 4.1.0 software. (**A**) The 3D structure of frog PrP with Trp6 and Leu6 alleles. (**B**) The 3D structure of frog PrP with Cys8 and Tyr8 alleles. (**C**) The 3D structure of frog PrP with Ser143 and Asn143 alleles. (**D**) The 3D structure of frog PrP with Thr207 and Ser207 alleles. (**E**) The 3D structure of frog PrP with Arg211 and Trp211 alleles. (**F**) The 3D structure of frog PrP with Leu241 and Phe241 alleles. The red box indicates the functional groups of the target amino acid. The green and gray dotted lines indicate hydrogen bonds. The green and gray numbers indicate the length of the hydrogen bond. The orange arrow indicates the region where the hydrogen bond length changed.

**Figure 4:**
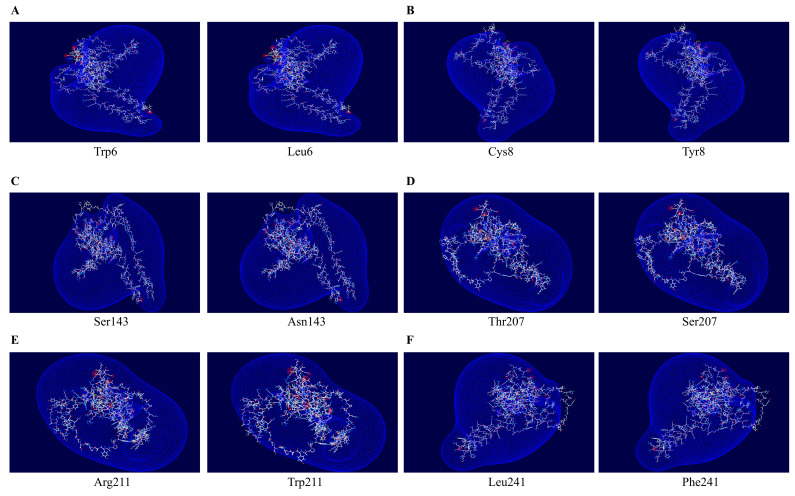
The electrostatic potential prediction of frog PrP according to six non-synonymous SNPs. (**A**) The electrostatic potential of frog PrP with Trp6 and Leu6 alleles. (**B**) The electrostatic potential of frog PrP with Cys8 and Tyr8 alleles. (**C**) The electrostatic potential of frog PrP with Ser143 and Asn143 alleles. (**D**) The electrostatic potential of frog PrP with Thr207 and Ser207 alleles. (**E**) The electrostatic potential of frog PrP with Arg211 and Trp211 alleles. (**F**) The electrostatic potential of frog PrP with Leu241 and Phe241 alleles. The color of the molecular surface indicates the electrostatic potential: blue: positive potential; red: negative potential.

To predict the frog PrP structural changes according to non-synonymous SNPs of the frog *PRNP* gene, we utilized ColabFold and Swiss-Pdb Viewer. Differences in the lengths of hydrogen bonds were noted between Ser143 and Asn143 and between Thr207 and Ser207 according to non-synonymous SNPs. The hydrogen bond length changed from 2.54 Å to 2.53 Å when Ser143 was replaced with Asn143. In addition, the hydrogen bond length changed from 2.77 Å to 2.64 Å when Thr207 was replaced with Ser207. Except for two non-synonymous SNPs, there were no changes in hydrogen bond length (Figure 3D). In addition, although changes in hydrogen bonds were observed due to non-synonymous SNPs, no significant changes in the electrostatic potential were observed for any of them (Figure 4).

### 3.5. Evaluation of Functional Alteration of Frog PrP According to Non-Synonymous SNPs

We estimated the functional alteration of frog PrP according to six non-synonymous SNPs using PolyPhen-2 Ver. 2.2.3, PANTHER Ver. 19.0, MutPred2 Ver. 2.0, and SIFT Ver. 6.2.1 software (Table 3). The c.17G>T (Trp6Leu) was predicted to be “Possibly Damaging” by PolyPhen-2 with a score of 0.816 and by PANTHER with a score of 361. MutPred2 predicted the Trp6Leu to be “Pathogenic” with a score of 0.613, and SIFT was estimated to be “Damaging” with a score of 0.00. The SNP c.23G>A (Cys8Tyr) was predicted to be “Probably Damaging” with a score of 0.969 by PolyPhen-2, “Benign” with a score of 0.232 by MutPred2, and “Damaging” with a score of 0.04 by SIFT. The SNP c.428G>A (Ser143Asn) was predicted to be “Possibly Damaging” with a score of 0.953 by PolyPhen-2, “Benign” with a score of 0.259 by MutPred2, and “Tolerated” with a score of 0.97 by SIFT. c.619A>T (Thr207Ser) was predicted to be “Benign” with a score of 0.002 by PolyPhen-2, “Benign” with a score of 0.102 by MutPred2, and “Tolerated” with a score of 0.78 by SIFT. c.631C>T (Arg211Trp) was predicted to be “Probably Damaging” by PolyPhen-2 with a score of 0.984 and ‘Possibly Damaging’ by PANTHER with a score of 361. MutPred2 predicted Arg211Trp to be “Benign” with a score of 0.477, and SIFT predicted it to be “Damaging” with a score of 0.00. The SNP c.721C>T (Leu241Phe) was predicted to be “Possibly Damaging” by PolyPhen-2 with a score of 0.915 and by PANTHER with a score of 361. MutPred2 predicted Leu241Phe to be “Pathogenic” with a score of 0.648, and SIFT predicted it to be “Damaging” with a score of 0.01.

### 3.6. In Silico Analysis of the Aggregation Propensity of the Frog PrP According to Non-Synonymous SNPs

To identify the aggregation propensity of the frog PrP, we performed an in silico analysis using AMYCO software. Except for the N143 allele, all non-synonymous SNPs were predicted to have scores identical to that of the wild-type PrP. Of note, the N143 allele had a higher score of 0.30 compared to that of the wild type (Table 3).

### 3.7. Multiple Sequence Alignments and Phylogenetic Analysis

To identify differences in the ORF of the frog *PRNP* gene compared with prion-related species, including prion-susceptible species (human, sheep, goat, cattle, and deer) and prion-resistant species (dog and horse), we analyzed the DNA sequences using ClustalW2 (Supplementary Figure S1). Among the prion-related species, Dybowski’s frog showed the highest ORF similarity with *Ovis aries* (sheep, 50.77%), followed by *Capra hircus* (goat, 50.49%), and *Bos taurus* (cattle, 49.86%).

We carried out multiple sequence alignments to identify the difference in the frog PrP compared to those of prion-related species (Supplementary Figure S2). Among prion-related species, Dybowski’s frog showed the highest PrP similarity with *Odocoileus hemionus* (Mule deer, 38.46%), followed by *Ovis aries* (sheep, 37.45%), and *Bos taurus* (cattle, 37.04%). Of note, Dybowski’s frog PrP showed low similarity with those of prion-related species (<40%). In addition, as described in previous studies on the African clawed and Dybowski’s frogs [27,41,42], Dybowski’s frog does not have tandem repeat domains. However, a partially conserved PrP^C^–PrP^Sc^ interaction region was observed (AGAAAVGV).

To analyze the evolutionary distances of the frog PrP sequence compared with those of prion-related species, a phylogenetic analysis was conducted using the MEGA X program (Supplementary Figure S3). Dybowski’s frog showed the closest evolutionary distance to dog, followed by sheep and goat. In contrast, horse exhibited the farthest evolutionary distance from Dybowski’s frog.

## 4. Discussion

Research on prion diseases has been conducted on various animals such as sheep, cattle, goats, deer, birds, and dogs, and major concerns of that research are the susceptibility and resistance to prion diseases according to genetic polymorphisms in the *PRNP* gene [5,12,21,43,44,45]. In previous studies, the risk of classical scrapie can be classified into five types according to the 136 (A>V), 154 (R>H), and 171 (R>Q, H) polymorphisms in the ovine *PRNP* gene in sheep [21]. In addition, in the *PRNP* gene of cattle, a 12 bp deletion in the promoter was associated with BSE susceptibility, and an additional 23 bp deletion in the same promoter showed the highest susceptibility to BSE [12]. In addition, dogs and horses were found to show high resistance to prion diseases based on the results of *PRNP* polymorphism studies [44,46]. Previous studies have shown that sheep and goats have 43 SNPs and 22 SNPs, respectively [47], while horses and dogs have 7 SNPs [44,46,48,49], and chickens have 0 SNPs [50]. This indicates that prion-susceptible animals such as sheep and goats have highly polymorphic *PRNP* gene, whereas prion-resistant animals such as horses, dogs, and chickens have low-polymorphism *PRNP* genes. Although this relationship is not well-established, to the best of our knowledge, our first report with a total of 34 SNPs in the frog *PRNP* gene (Figure 1, Table 1) suggests frogs may be prion-susceptible animals.

In sheep, several well-documented polymorphisms in the *PRNP* coding region, such as A136V, R154H, and Q171R, have been strongly associated with scrapie susceptibility [5,47]. Similarly, E211K mutation in cattle has been implicated in BSE [51,52]. These mutations highlight species–specific differences in the genetic susceptibility to transmissible spongiform encephalopathies (TSEs). However, the *PRNP* gene sequence in frogs shows less than ~50% similarity to prion-related species at the DNA level and less than ~40% similarity at the PrP protein level (Supplementary Figures S1 and S2). Due to this low sequence similarity, direct comparisons of prion-disease-associated mutations between frogs and mammals, such as sheep and cattle, are limited. Despite these limitations, our findings provide an initial framework for understanding *PRNP* polymorphisms in amphibians and lay the groundwork for future studies to explore evolutionary and functional differences in prion susceptibility across species. Further verification is required to determine whether the genetic polymorphisms in frogs can act as prion seeds, either through PrP^Sc^ detection or brain homogenate infection experiments in the future.

Specific SNPs within the *PRNP* gene are known to significantly impact the structure and function of the PrP [5,53,54]. To evaluate if the polymorphisms affect the structure and function of PrP, we conducted an in silico analysis (Table 3, Figure 3 and Figure 4). Although we identified numerous SNPs in the frog *PRNP* gene, no insertion/deletion polymorphisms were observed. In this study, the W6L and C8Y SNPs, located in the signal peptide region, were predicted to have damaging effects by potentially disrupting the proper intracellular localization of PrP (Table 3). Furthermore, the S143N SNP was predicted to have functional pathogenicity by PolyPhen-2 and was found to increase in amyloidogenic propensity based on AMYCO (Table 3). This substitution may act as a key factor that increases the likelihood of pathological PrP conversion. Polymorphisms at codons 207 and 211 located in the globular domain are expected to significantly alter the secondary structure and stability of PrP [55]. Notably, this domain is a critical region where numerous pathogenic SNPs have been identified in humans, underscoring the need for further investigation [56,57]. In addition, residues 143 and 207 are associated with weakened hydrogen bonding, which may render these regions structurally vulnerable and facilitate transitions to pathological conformations (Figure 3). These genetic polymorphisms may be especially relevant to the conversion of normal PrP^C^s into pathogenic PrP^Sc^s. Therefore, further studies using in vitro and in vivo systems with these residues are essential for understanding the pathological mechanisms underlying prion diseases in amphibians.

Our findings do not provide direct evidence to confirm the hypothesis that prion seeds derived from animals at lower evolutionary stages are the cause of scrapie. The complexity of prion disease transmission pathways, involving multiple environmental and biological factors, underscores the challenges in identifying definitive sources of prion diseases such as scrapie [24,58]. Furthermore, the absence of reported causal mutations of prion seed in non-human hosts does not conclusively support the hypothesis that these animals serve as a reservoir or source for prion transmission. Despite these limitations, our study offers valuable insights into the potential evolutionary connections and molecular characteristics of prion seeds in amphibians. These findings contribute to the foundational understanding necessary for further exploration of prion diseases across diverse species.

While recent trends in prion research have focused primarily on mammals, relatively little research has been conducted on amphibians [41,42]. A previous study has compared the amino acid sequences of the *PRNP* gene of the frog species *Xenopus laevis* with those of other species. As a result, the unique palindrome sequence and the absence of octapeptide repeat regions in frog were revealed. In addition, the nuclear magnetic resonance (NMR) structure of frog PrP has revealed a well-preserved structure of three α-helixes and two β-sheets, similar to human PrP, but both beta sheets are longer than those in human PrP. The most recent study of frog prion compared DNA sequences of the *PRNP* gene among seven frog species, including novel identified *PRNP* gene sequences for Dybowski’s frog (*Rana dybowskii*) and the American bullfrog (*Lithobates catesbeianus*). Of note, seven frog species were revealed to lack the octapeptide repeat sequence and have partially conserved palindromic motifs (A**AA*G*), which is critical for PrP^C^–PrP^Sc^ interactions. In addition, the aggregation tendency of PrP was analyzed in seven frog species, among which high aggregation propensity potential was observed in High Himalayan frogs (*Nanorana parkeri*) [27]. These results suggest that some frog species exhibit a naturally high potential for amyloid formation by AMYCO analysis. These results indicate the importance of the investigation of prion research in frog species. In addition, since SNPs in the *PRNP* gene in frog species may generate pathological PrP^Sc^s, further investigation between the prion disease and frog *PRNP* SNPs found in this study is highly desirable.

The American bullfrog is an invasive species introduced to the Republic of Korea in 1958. It is also consumed as a health food, similar to Dybowski’s frog. Thus, the future study of genetic polymorphisms in the American bullfrog is important. Given the observed prion sensitivity characteristic in frogs such as high polymorphism rates, further diagnostic studies are essential to confirm whether pathological PrP^Sc^s are produced in frogs.

## 5. Conclusions

In the present study, we identified 34 novel polymorphisms in the *PRNP* gene of Dybowski’s frog, including 6 non-synonymous SNPs and 28 synonymous SNPs. Among the non-synonymous SNPs, W6L and C8Y variations were predicted to impart greater damage due to being located in the signal peptide region. Notably, the S143N polymorphism was expected to have a damaging effect through PolyPhen-2 and to increase amyloid propensity. Interestingly, the alteration in length of hydrogen bonds was observed at codons 143 and 207 according to non-synonymous SNPs, while no alteration of electrostatic potential was observed in six non-synonymous SNPs. In addition, genetic polymorphisms at codons 211 and 241 were predicted to be damaging and are closely related to pathogenic mutations of human PrP. The *PRNP* gene and PrP sequences of frogs exhibited low homology with those of prion-related mammals. To the best of our knowledge, this is the first report on *PRNP* polymorphisms in frogs, underscoring their pathogenic potential using in silico analytic models.

## Figures and Tables

**Table 1:** Genotype and allele frequencies of the prion protein gene (*PRNP*) polymorphisms in 194 frogs.

Polymorphisms	Genotype Frequency (%)			Allele Frequency (%)	
	M/M	M/m	m/m	M	m
c.17G>T	75.77	3.61	20.62	77.58	22.42
c.23G>A	92.27	6.70	1.03	95.62	4.38
c.30C>T	76.29	9.28	14.43	80.93	19.07
c.42C>T	86.60	6.18	7.22	89.69	10.31
c.63C>G,A	63.40	8.76	27.84	67.78	32.22
c.111C>T	98.96	0.52	0.52	99.23	0.77
c.120C>T	69.59	22.16	8.25	80.67	19.33
c.177A>G	96.39	2.58	1.03	97.68	2.32
c.198A>T	97.94	2.06	0	98.97	1.03
c.237C>T	75.26	15.46	9.28	82.99	17.01
c.252C>T	96.91	3.09	0	98.45	1.55
c.291C>T	98.97	1.03	0	99.48	0.52
c.303G>A	86.08	10.31	3.61	91.24	8.76
c.321C>T	76.29	15.98	7.73	84.28	15.72
c.339T>C	83.50	13.92	2.58	90.46	9.54
c.372C>T	64.43	35.05	0.52	81.96	18.04
c.378T>A	60.31	12.37	27.32	66.49	33.51
c.381A>G	72.16	13.92	13.92	79.12	20.88
c.428G>A	98.45	1.55	0	99.23	0.77
c.492C>A	91.75	7.22	1.03	95.36	4.64
c.525T>C	98.97	1.03	0	99.48	0.52
c.540T>A	62.37	11.86	25.77	68.30	31.70
c.549T>A	70.62	18.56	10.82	79.90	20.10
c.558A>T	97.42	2.58	0	98.71	1.29
c.603C>T	89.69	8.25	2.06	93.81	6.19
c.610A>C	90.72	6.70	2.58	94.07	5.93
c.619A>T	92.78	3.61	3.61	94.59	5.41
c.627C>T	94.33	5.67	0	97.16	2.84
c.631C>T	91.24	8.76	0	95.62	4.38
c.691C>T	97.94	2.06	0	98.97	1.03
c.693G>A	89.69	8.76	1.55	94.07	5.93
c.717C>G	97.42	2.58	0	98.71	1.29
c.721C>T	78.86	7.22	13.92	82.47	17.53
c.735C>A	94.85	5.15	0	97.42	2.58

M/M, major homozygote; M/m, heterozygote; m/m, minor homozygote; M, major allele; m, minor allele.

**Table 2:** Haplotype frequencies of 34 genetic polymorphisms of the *PRNP* gene in frogs.

-	Haplotypes	Frequency (*n* = 388)
Haplotype 1	GGCCGCCAACCCGCTCTAGCTTTACAACCCGCTC	49 (0.126)
Haplotype 2	TGCCCCTAACCCGCTCTAGCTTTACAACCCGCCC	43 (0.111)
Haplotype 3	GGCCCCCAACCCGCTCTAGCTTTACAACCCGCCC	34 (0.087)
Haplotype 4	TGCCCCCAACCCGCTCTAGCTTTACAACCCGCCC	19 (0.050)
Haplotype 5	GGCCGCCAATCCACCCAAGCTAAATCTCCCGCCC	12 (0.031)
Haplotype 6	GGTTCCCAATCCGTTCAGGCTAAACAACCCACCC	12 (0.031)
Haplotype 7	GGCCCCCAACCCGCTCTAGCTATACAACCCGCCC	11 (0.030)
Haplotype 8	TGCCCCTAACCCGCTTTAGCTTTACAACCCGCCC	10 (0.025)
Haplotype 9	GGCCGCCAACCCGCTCTAGCTTTACAACCCGCCC	10 (0.025)
Haplotype 10	GGCCCCCAACCCGCTCTAGATTTACAACCCGCCC	8 (0.020)
Haplotype 11	GGCCGCTAACCCGCTCTAGCTTTACAACCCGCTC	7 (0.018)
Haplotype 12	GGCCCCTAACCCGCTCTAGCTTTACAACCCGCCC	6 (0.016)
Haplotype 13	GGCCCCCAACCCGCTTTAGATTTACAACCCGCCC	6 (0.016)
Haplotype 14	GGCCCCCAACCCGCTTTAGCTTTACAACCCGCCC	6 (0.016)
Haplotype 15	GGTCCCCAACCCGTTCAGGCTTTACAACCCGCCA	6 (0.015)
Haplotype 16	GGTTCCCAACCCGCTCAAGCTATACAACCCGCCC	6 (0.015)
Haplotype 17	GGTCCCCAACCCGTTCAGGCTAAACAACCCGCCC	5 (0.013)
Haplotype 18	TGCCCCCAACCCGCTTTAGCTTTACAACCCGCCC	5 (0.012)
Haplotype 19	GGCCCCCAACCCGCTCAAGCTATACAACCCGCCC	4 (0.011)
-	Others *	129 (0.332)

* Others contain rare haplotype frequencies of <0.01.

**Table 3:** In silico prediction of the effects of non-synonymous SNPs in the *PRNP* gene of frogs.

Variations	Methods	Score	Prediction
c.17G>T (W6L)	PolyPhen-2	0.816	Possibly Damaging
	PANTHER	361	Possibly Damaging
	MutPred2	0.613	Pathogenic
	SIFT	0.00	Damaging
	AMYCO	0.29	Identical
c.23G>A (C8Y)	PolyPhen-2	0.969	Probably Damaging
	PANTHER	-	Not scored
	MutPred2	0.232	Benign
	SIFT	0.04	Damaging
	AMYCO	0.29	Identical
c.428G>A (S143N)	PolyPhen-2	0.953	Possibly Damaging
	PANTHER	-	Not scored
	MutPred2	0.259	Benign
	SIFT	0.97	Tolerated
	AMYCO	0.30	Increase
c.619A>T (T207S)	PolyPhen-2	0.002	Benign
	PANTHER	-	Not scored
	MutPred2	0.102	Benign
	SIFT	0.78	Tolerated
	AMYCO	0.29	Identical
c.631C>T (R211W)	PolyPhen-2	0.984	Probably Damaging
	PANTHER	361	Possibly Damaging
	MutPred2	0.477	Benign
	SIFT	0.00	Damaging
	AMYCO	0.29	Identical
c.721C>T (L241F)	PolyPhen-2	0.915	Possibly Damaging
	PANTHER	361	Possibly Damaging
	MutPred2	0.648	Pathogenic
	SIFT	0.01	Damaging
	AMYCO	0.29	Identical

## Data Availability

Access to original data will be granted by the original author upon reasonable request.

## Supplementary Materials

The following supporting information can be downloaded at: https://www.mdpi.com/article/10.3390/ani15020220/s1, Figure S1: The comparison of the ORF of the frog *PRNP* gene with those of prion-related species; Figure S2: The comparison of the amino acid sequences of the frog PrP with those of prion-related species; Figure S3: The phylogenetic tree of PrPs from eight species was constructed using the Molecular Evolutionary Genetics Analysis (MEGA) X program with the maximum likelihood method (bootstrap 3000 replicates); Table S1: Collection of Dybowski’s frog samples from various regions.
