# Supplementary material for: Identification of the Highly Polymorphic Prion Protein Gene (PRNP) in Frogs (Rana dybowskii)
Source: Animals (Basel). 2025 Jan 15;15(2):220. doi: 10.3390/ani15020220 (PMC11758322; doi:10.3390/ani15020220)
Supplement: Supplementary file 1 [file animals-15-00220-s001.zip › animals-3406752-supplementary.pdf]

(A)

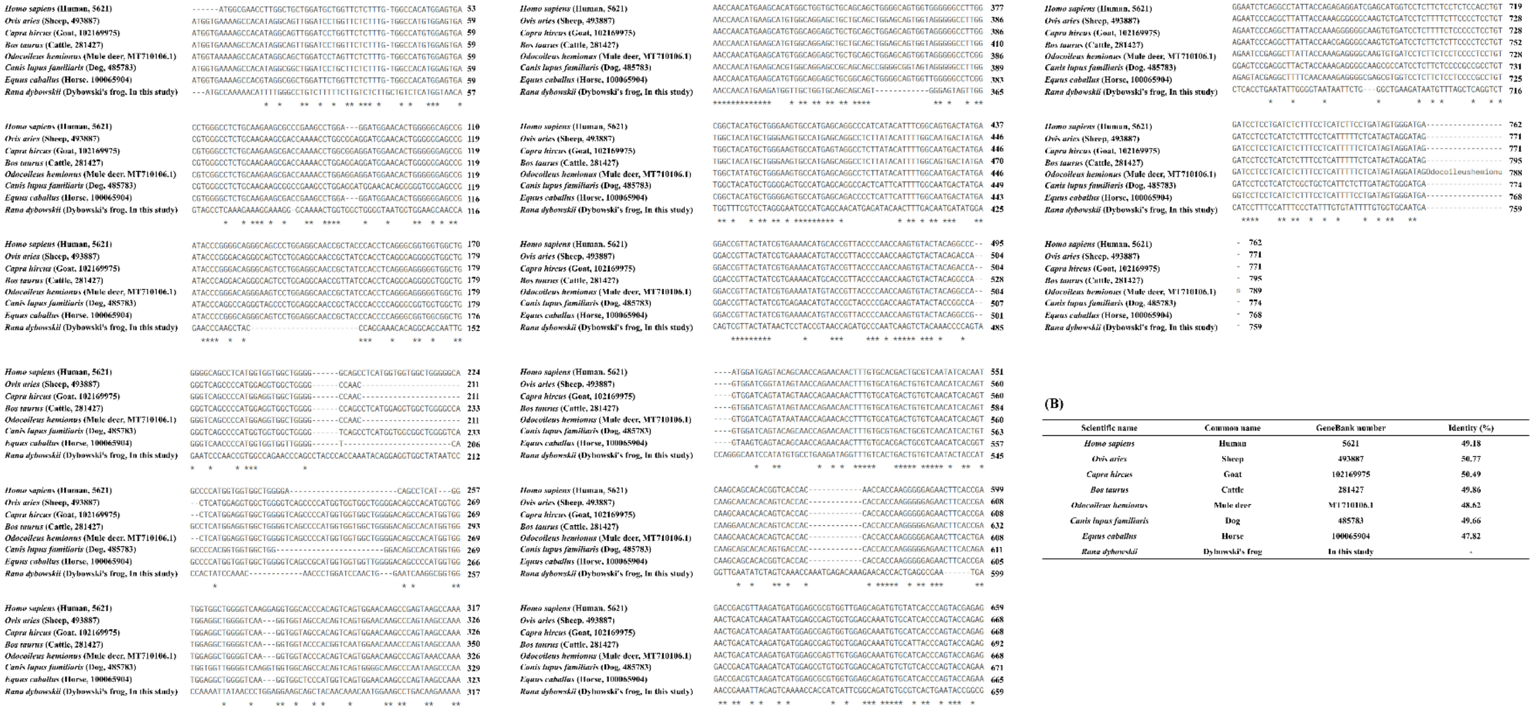

(B)

Scientific name	Common name	GeneBank number	Identity (%)
Homo sapiens	Human	5621	49.18
Ovis aries	Sheep	493887	50.77
Capra hircus	Goat	102169975	50.49
Bos taurus	Cattle	281427	49.86
Odocoileus hemionus	Mule deer	MT710106.1	48.62
Canis lupus familiaris	Dog	485783	49.66
Equus caballus	Horse	100065904	47.82
Rana dybowskii	Dybowski's frog	In this study	-

Supplementary Figure 1

Supplementary Figure 1

The comparison of the ORF of the frog *PRNP* gene with those of prion-related species. (A) Multiple sequence alignment of ORF of the *PRNP* gene by ClustalW2. (B) Detailed information on the sequence similarity of ORF of the *PRNP* gene sequences in eight species. The ORF alignment includes *Homo sapiens* (human, Gene ID: 5621), *Ovis aries* (sheep, Gene ID: 493887), *Capra hircus* (goat, Gene ID: 102169975), *Bos taurus* (cattle, Gene ID: 281427), *Odocoileus hemionus* (mule deer, Gene ID: MT710106.1), *Canis lupus familiaris* (dog, Gene ID: 485783), *Equus caballus* (horse, Gene ID: 100065904), *Rana dybowskii* (Dybowski's frog, Gene ID: In this study). Asterisks denote identical positions among species.

(A)

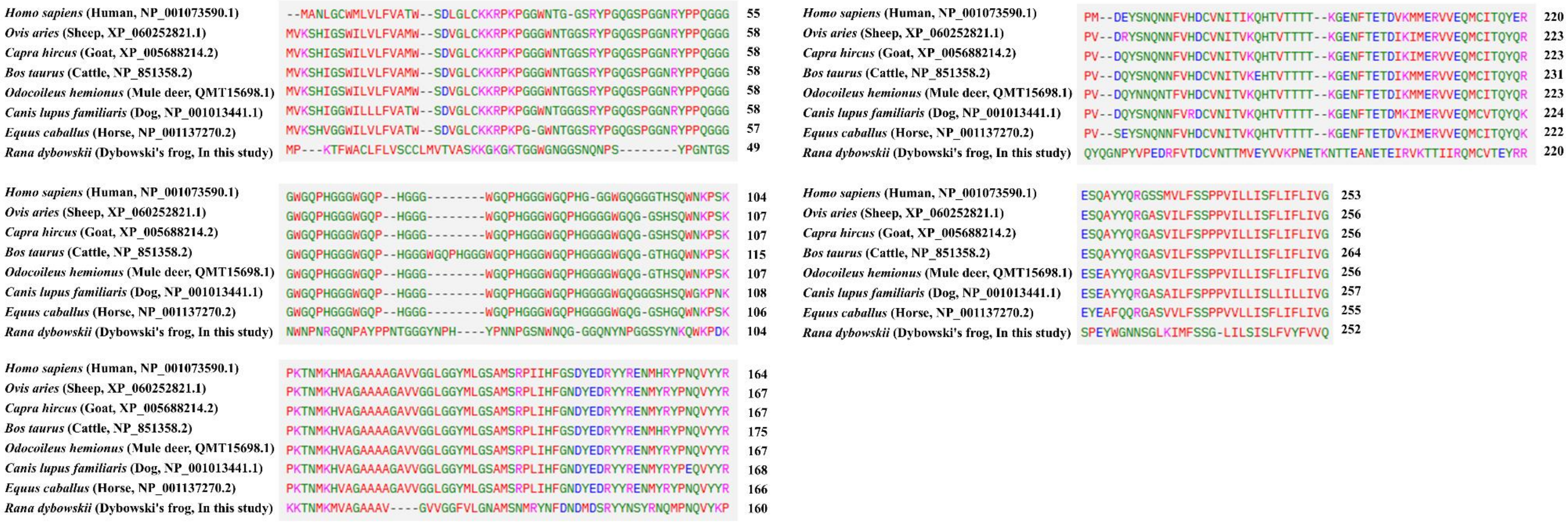

(B)

Scientific name	Common name	GeneBank number	Identity (%)
Homo sapiens	Human	NP_001073590.1	33.33
Ovis aries	Sheep	XP_060252821.1	37.45
Capra hircus	Goat	XP_005688214.2	37.02
Bos taurus	Cattle	NP_851358.2	37.04
Odocoileus hemionus	Mule deer	QMT15698.1	38.46
Canis lupus familiaris	Dog	NP_001013441.1	36.86
Equus caballus	Horse	NP_001137270.2	35.04
Rana dybowskii	Dybowski's frog	In this study	-

Supplementary Figure 2

Supplementary Figure 2

The comparison of the amino acid sequences of the frog PrP with those of prion-related species. (A) Multiple sequence alignment of the PrP sequences by ClustalW2. (B) Detailed information on the sequence similarity of PrP sequences in eight species. The PrP sequence alignment includes *Homo sapiens* (human, Protein ID: NP_001073590.1), *Ovis aries* (sheep, Protein ID: XP_060252821.1), *Capra hircus* (goat, Protein ID: XP_005688214.2), *Bos taurus* (cattle, Protein ID: NP_851358.2), *Odocoileus hemionus* (mule deer, Protein ID: QMT15698.1), *Canis lupus familiaris* (dog, Protein ID: NP_001013441.1), *Equus caballus* (horse, Protein ID: NP_001137270.2), *Rana dybowskii* (Dybowski's frog, Protein ID: in this study). Four colors represent the chemical properties of amino acids (magenta, basic; green: hydroxyl, sulphhydryl, amine and glycine; red, small and hydrophobic; blue: acid).

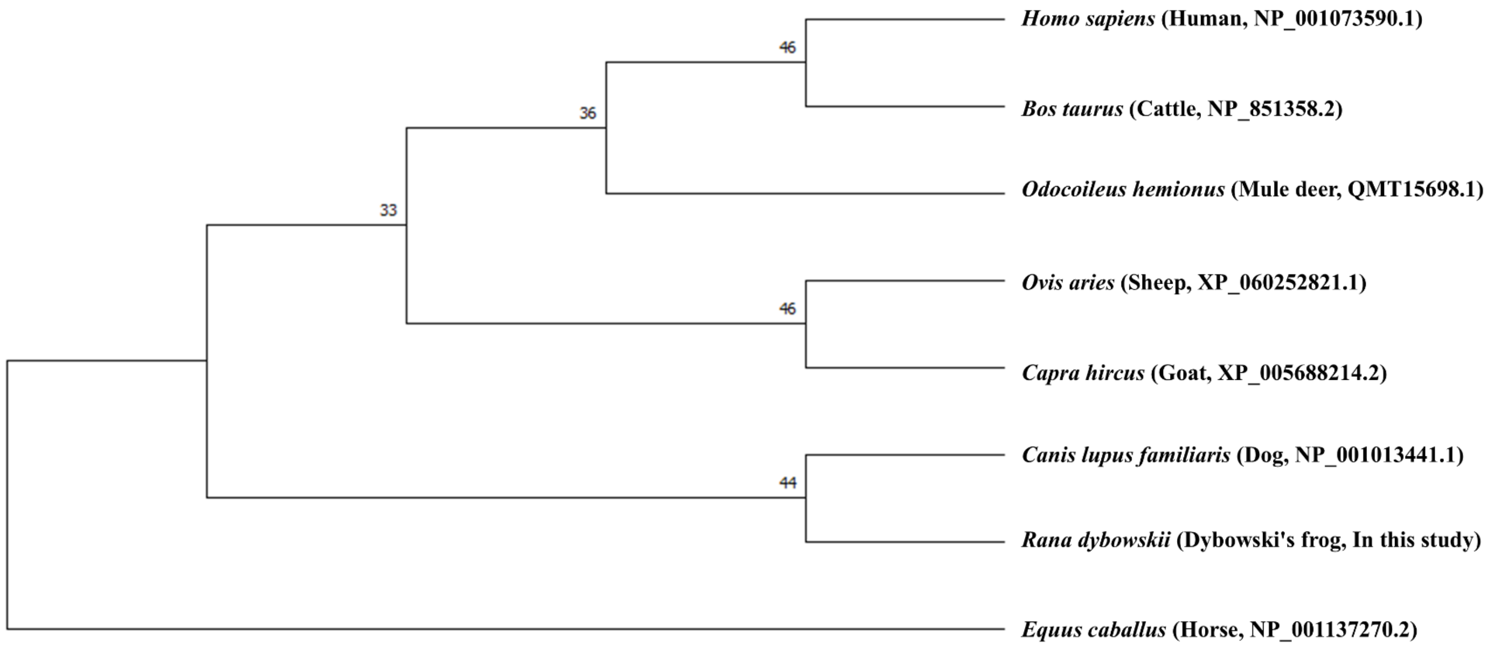

Supplementary Figure 3

Supplementary Figure 3

The phylogenetic tree of PrPs from eight species was constructed using the Molecular Evolutionary Genetics Analysis (MEGA) X program with the maximum likelihood method (bootstrap 3,000 replicates). The phylogenetic analysis includes *Homo sapiens* (human, Protein ID: NP_001073590.1), *Ovis aries* (sheep, Protein ID: XP_060252821.1), *Capra hircus* (goat, Protein ID: XP_005688214.2), *Bos taurus* (cattle, Protein ID: NP_851358.2), *Odocoileus hemionus* (mule deer, Protein ID: QMT15698.1), *Canis lupus familiaris* (dog, Protein ID: NP_001013441.1), *Equus caballus* (horse, Protein ID: NP_001137270.2), *Rana dybowskii* (Dybowski's frog, Protein ID: in this study). The branch lengths were calculated based on the number of amino acid substitutions per site.

Supplementary Table 1. Collection of Dybowski's frog samples from various regions.

Regions		Number of samples (n)
Province	City	
Gangwon-do	Gangneung-si	79
Gangwon-do	Chuncheon-si	47
Gyeonggi-do	Namyangju-si	34
Gangwon-do	Chuncheon-si	34
